# Evaluation of Serum Lysophosphatidic Acid and Lysophosphatidylcholine Levels in Major Depressive Disorder Patients

**DOI:** 10.7759/cureus.12388

**Published:** 2020-12-30

**Authors:** Sumaia Riya, Sharmin Sultana, Sohel Daria, Maliha A Proma, Mohiuddin Ahmed Bhuiyan, Md Ahsanul Haque, Md Rabiul Islam

**Affiliations:** 1 Department of Pharmacy, University of Asia Pacific, Dhaka, BGD

**Keywords:** lysophosphatidic acid, lpa, lysophosphatidylcholine, lpc, major depressive disorder, mdd

## Abstract

Background

Major depressive disorder (MDD) is a heterogeneous condition featured with a continuous low mood, feeling of sadness, lack of interest to perform daily activities. Genetic, physiological, biological, social, and environmental factors are associated with the pathophysiology of depression. Though several human studies failed to identify the suitable biological markers for depression, some animal studies showed phospholipids play a vital role in the alteration of emotion. Thus, the current study aimed to measure the serum levels of lysophosphatidic acid (LPA) and lysophosphatidylcholine (LPC) in MDD patients and healthy controls (HCs) to explore their roles and relationship with depression.

Methods

This case-control study enrolled 53 MDD patients and 50 HCs matched by age, gender, and body mass index. Based on the diagnostic and statistical manual of mental disorders, 5^th^ edition, a qualified psychiatrist diagnosed patients and assessed HCs. We applied the Hamilton depression rating scale (Ham-D) to measure the severity of depression. We used enzyme-linked immunosorbent assay kits to measure serum lysophosphatidic acid and lysophosphatidylcholine levels.

Results

We found no alterations of these parameters in serum levels of MDD patients compared to HCs. We also observed a significant positive correlation between LPA and LPC levels in MDD patients. Moreover, the present study showed no significant associations between target markers and either diagnosis of depression or Ham-D scores, or management of depression.

Conclusion

The present study suggests that LPA and LPC levels probably would not serve as potential biomarkers of MDD. Thus, we recommend further studies with large and more homogeneous populations to explore the exact relationship between serum lipids and MDD.

## Introduction

Major depressive disorder (MDD) is a complex psychiatric condition; it is unlikely to identify a single causal factor for it [1]. Genetic, biological, psychological, and environmental issues are involved in depression [2-6]. As MDD is highly comorbid, the neurobiological basis of depression needs to be known for efficient diagnosis and treatment [7]. Previous studies suggest the malfunction in neuronal proteins and peptides are involved in the pathophysiology of MDD [8-10]. Brain tissue and neural membrane contain a relatively high amount of glycerophospholipids [11]. These glycerophospholipids act as signaling molecules and are responsible for the formation of cell membranes [12]. As signaling molecules, they can easily promote anxiety and depressive disorders [7]. Lysophosphatidic acid (LPA) is one of the most significant reactive phospholipid species [13]. LPA is synthesized and metabolized in a large number of metabolic pathways. Among all, the enzymatic action of autotoxin is the most crucial pathway [14]. The second important pathway is to obtain LPA from membrane phospholipids with the help of phospholipase enzymes [15]. There are many other additional pathways for producing LPA, particularly by acyl-transferase enzymes [16]. MDD is one of the most common psychiatric illness that has created an immense burden in the community. For clinical purposes, there are no established biochemical markers for this disorder though LPA is a potent bioactive lipid mediator with various biological characteristics [17, 18]. The LPA1-receptor knockout mice showed altered emotional behavior due to the change of LPA receptors [19]. Therefore, LPA is one of the essential regulators of behavior and may involve in the pathophysiology of MDD.

Lysophosphatidylcholine (LPC) is another kind of phospholipid that has a significant role in the neuronal mechanisms underlying the pathophysiology of several psychiatric disorders [20]. As a component of serum lipoprotein, LPC is a water-soluble amphiphilic lysophospholipid molecule [21]. In cell-biology, destabilization of the lysosomal process is troublesome for organelle and living cells [22]. LPC is one of the leading phospholipids elements of oxidized low-density lipoprotein (Ox-LDL) produced from the biochemical transformation of phosphatidylcholine [23]. The promptness of diverse signaling pathways is attached to the production of oxidative stress and inflammatory responses by LPC [24]. Studies suggest that polyunsaturated fatty acids could involve in the pathophysiology of MDD [25]. Based on the above findings, LPA and LPC might impact brain functioning and emotional behaviors. The role of LPA and LPC in depression is yet unclear though many studies conducted in these regards. Different researchers have different opinions regarding the association of LPA and LPC with depression [26, 27]. Therefore, the present study aimed to analyze serum LPA and LPC levels in MDD patients and their corresponding controls to decide the affiliation between the changed LPA and LPC levels and the seriousness of MDD.

## Materials and methods

Study population

To achieve a confidence level of 95% with a ±5% margin of error, we needed to recruit 87 participants if the population proportion is 6%. The present study included 103 subjects (53 MDD patients and 50 controls) for this case-control study based on the above assumption from September 2019 to July 2020. The patients enrolled from Bangabandhu Sheikh Mujib Medical University (BSMMU), Dhaka, Bangladesh. We recruited the matched (age, gender, and BMI) healthy controls (HCs) from the different locations of Dhaka city. A specialized psychiatrist diagnosed and evaluated both the cases and controls according to the diagnostic and statistical manual of mental disorders, 5th edition (DSM-5). All the participants performed a detailed neurological and physical screening for the coexistence of other complications. The severity scores of depression were measured using the 17-items Hamilton depression rating scale (Ham-D). Ham-D items were put together into the following segments: core (items 1, 2, 7, 8, 10, 13), sleep (items 4, 5, 6), activity (items 7, 8), psychic anxiety (items 9, 10), somatic anxiety (items 11, 12, 13), delusion (items 2, 15) [28]. We excluded the subjects with a previous history of cardiovascular disease, epilepsy, hypertension, liver or kidney failure, and patients treated with any medication that could potentially modify the concentration of serum LPA and LPC levels. Also, we eliminated the patients who were suffering from mental retardation and other comorbid psychiatric illnesses. Additional exclusion criteria were participants with substance abuse or dependency, severe organic conditions, abnormal body mass index (BMI), and the presence of infectious diseases. We used a structured-predesigned questionnaire to record the socio-demographic and different biophysical characteristics.

Blood sample collection and processing

After overnight fasting of 10-12 hours, a professional phlebotomist collected blood samples (5mL) of study participants from the cephalic vein by using a plastic syringe fitted with a stainless-steel needle. The collected blood samples were placed into falcon tubes and were allowed to clot for an hour at room temperature without any agitation. The serum samples were extracted from the collected blood samples by centrifugation at 1000 x g for 15 minutes at room temperature. We stored the separated serum samples immediately at -80° C until being used for further analysis.

Quantification of serum LPA and LPC

We used commercially available enzyme-linked immunosorbent assay (ELISA) kits (Abbexa Ltd., UK) to quantify the serum LPA and LPC levels. That is a competitive binding ELISA technology where antibodies specific to LPA and LPC were pre-coated onto the 96 well-plates. Briefly, we took 50μL of standard solution and serum sample on a 96 well-plate and gently shaken the plate to mix the contents thoroughly. A 50μL of detection reagent-A was added to each well and gently shaken the plates for proper mixing. The plates were then sealed and incubated at 37° C temperature for 1 hour. Then discarded the liquid and washed the plates properly using wash buffer. After that, 100μL of detection reagent B was dispensed into each well with gentle mixing and sealed the plates for 30 min incubation at 37° C. After the repeated washing process, added a 90μL tetramethylbenzidine (TMB) substrate to each well. The plates were incubated again for 15 min at 37° C. Finally, we stopped the reaction by adding a 50μL of stop solution and took the absorbances immediately at 450nm. From these absorbance value, we calculated the serum LPA and LPC levels and presented as μmol/L. There was no cross-reactivity with other neurotrophic mediators. The coefficient of variance (CV) for intra-assay and inter-assay was <10% and <12%, respectively. The assay sensitivity for serum LPA and LPC were <52.7 ng/mL and <92.4 ng/mL, respectively.

Statistical analysis

We carefully checked and verified all the collected data to detect errors and rectified them accordingly. Then we performed the coding, classification, and tabulation of data. We applied the independent sample t-test for the continuous variables and Fisher’s exact test and categorical variables. We also conducted descriptive statistics for the socio-demographic characteristics of the study participants. In this research, we used the box plot and scatter plot graphs to showcase the study findings. We applied the statistical package for social sciences (SPSS), version 25.0 for dada analyses (IBM Inc., Armonk, USA). We considered statistically significant results with p-values less than 0.05.

## Results

We observed that both the cases and controls were similar in terms of their descriptive information, as presented in Table 1.

**Table 1:** Socio-demographic characteristics of the study population MDD - major depressive disorder; BMI - body mass index; CED - chronic energy deficiency; KBDT - kilo Bangladeshi taka; SEM - standard error mean

Variables	MDD patients (n=53) mean ± SEM	Healthy controls (n=50) mean ± SEM	p-value
Age	31.25 ± 1.20	34.53 ± 1.60	0.109
18-24	16 (30%)	11 (22%)	
25-34	17 (32%)	23 (46%)	
35-44	14 (27%)	13 (26%)	
45-60	6 (11%)	3 (6%)	
Gender			0.472
Male	22 (41%)	17 (34%)	
Female	31 (59%)	33 (66%)	
BMI (kg/m^2^)	24.15 ± 0.47	25.66 ± .073	0..263
Below 18.5 (CED)	4 (8%)	2 (4%)	
18.5–25 (normal)	34 (64%)	27 (54%)	
Above 25 (obese)	15 (28%)	21 (42%)	
Monthly income (KBDT)	57.27 ± 2.47	60.35 ± 3.25	0.462
Below 30	3 (6%)	5 (10%)	
30–60	23 (43)	18 (36%)	
61–90	19 (36)	12 (24%)	
Above 90	8 (15)	15 (30%)	
Education level			0.237
Illiterate	1 (2%)	5 (10%)	
Primary level	6 (11%)	1 (2%)	
Secondary level	29 (55%)	28 (56%)	
Graduate and above	17 (32%)	16 (32%)	
Occupation			0.253
Service	9 (17%)	4 (8%)	
Business	4 (8%)	8 (16%)	
Jobless	7 (13%)	9 (18%)	
Others	33 (62%)	29 (58%)	
Economic status			0.332
Low	13 (25%)	13 (26%)	
Medium	34 (64%)	27 (54%)	
High	6 (11%)	10 (20%)	
Smoking habit			0.526
Yes	11 (21%)	9 (18%)	
No	42 (79%)	41 (82%)	

**Figure 1:**
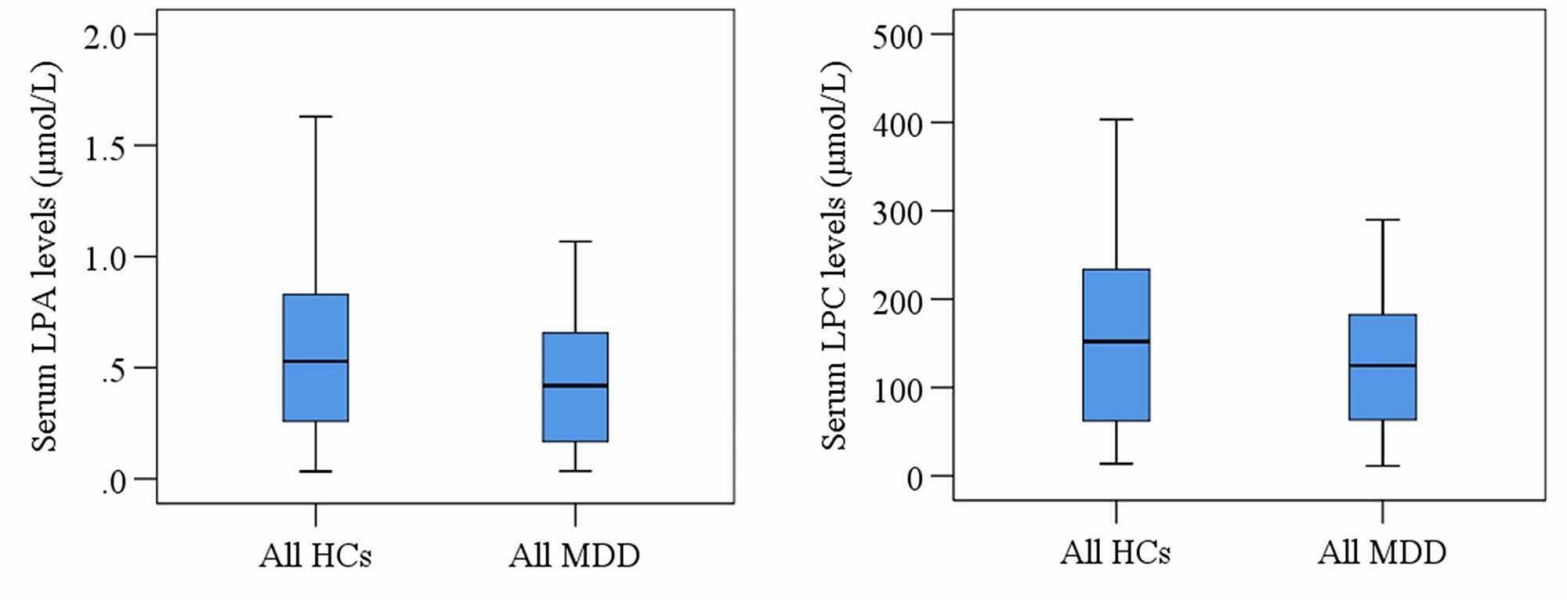
The box-plot graph depicts no significant changes in serum LPA and LPC among all of the study population between HCs and MDD patients LPA - lysophosphatidic acid; LPC - lysophosphatidylcholine; HCs - healthy controls; MDD - major depressive disorder

According to the present study, most of the MDD patients were females with normal BMI. MDD patients and their comparing controls were alike with regard to age (patients: 31.25 ± 1.20, controls: 34.53 ± 1.60, p=0.109), BMI (patients: 24.15 ± 0.47, controls: 25.66 ± 0.073, p=0.263), and sex (male/female: 22/31, 17/33 patients and controls in a corresponding manner, p=0.472). The non-smoker population with a medium economic impression was predominant in the present study. Following the past studies, the present study observed most of the MDD patients were in their third and fourth decades of life. Table 2 and Figure 1 demonstrated the clinical and laboratory findings of the study populations.

**Table 2:** Clinical characteristics and laboratory findings of the study population MDD - major depressive disorder; Ham-D - Hamilton depression rating scale; LPA - lysophosphatidic acid; LPC - lysophosphatidylcholine; SEM - standard error mean

Variables	MDD patients (n=53) mean ± SEM	Healthy controls (n=50) mean ± SEM	p-value
Ham-D score	15.90 ± 0.57	3.61 ± 0.44	<0.001
Serum LPA (μmol/L)	0.59 ± 0.09	0.66 ± 0.08	0.543
Male	0.58 ± 0.15	0.59 ± 0.10	0.991
Female	0.60 ± 0.12	0.70 ± 0.11	0.552
Serum LPC (μmol/L)	161.30 ± 22.96	167.59 ± 22.16	0.790
Male	192.10 ± 42.39	149.72 ± 42.39	0.264
Female	139.44 ± 25.21	176.79 ± 21.82	0.413

**Figure 2:**
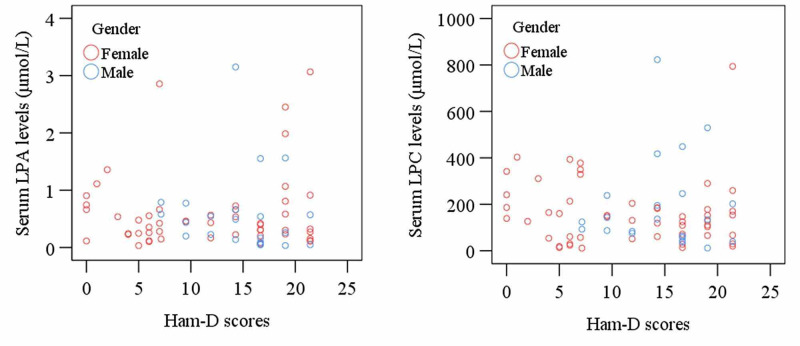
Scatter plot indicates no significant correlations of serum LPA and LPC versus Ham-D scores in MDD patients LPA - lysophosphatidic acid; LPC - lysophosphatidylcholine; Ham-D - Hamilton depression rating scale; MDD - major depressive disorder

Serum LPA and LPC levels did not show any significant changes in MDD patients compared to HCs (p>0.05). Moreover, serum LPA and LPC concentrations did not correlate with age, gender, and BMI in the patient group (Figure 2).

Significant positive correlations were observed between serum LPA and LPC levels in all MDD patients (r=0.739; p<0.001), male MDD patients (r=0.937; p<0.001), and female MDD patients (r=0.629; p<0.001). The present study obtained no significant correlation between LPA or LPC levels and Ham-D scores.

## Discussion

To the best of our knowledge, this is the first report concerning serum LPA and LPC in MDD patients from Bangladesh. The diagnosis of depression mainly depends on the structured clinical interview, and a reliable marker is not established yet for clinicians [17]. Here, we evaluated whether LPA or LPC levels could serve as an early risk assessment marker for MDD. Moreover, a study reported reduced serum autotaxin levels in MDD patients due to an impaired LPA axis function in the brain [26]. Liu et al. observed higher plasma LPC levels in MDD patients. Also, they found a significant positive correlation between these elevated LPC levels and the severity of depression [27]. Kim et al. also observed the decreased serum LPC levels in drug-treated or drug-naïve MDD patients compared to HCs [26]. A study reported lowered serum LPC levels in depression; others contrasted the assumption [26-29]. Thus, we verified the hypothesis that altered serum LPA and LPC levels are associated with the pathophysiology of MDD that could serve as early risk assessment or diagnostic purpose of depression. Despite the logic of our proposition, we observed that no significant alterations of serum LPA and LPC levels were happened in MDD patients compared to HCs, and that is why these phospholipids are unlikely to serve as early risk assessment markers of MDD. The present study is consistent with the past results where Gotoh et al. found no association of cerebrospinal fluid and plasma LPA levels with MDD [29]. The biological system obtained LPA from LPC with the enzyme autotoxin displays intriguing cell biology via G protein-coupled receptors. On the other hand, docosahexaenoic acid (DHA)-containing LPC performs efficient transporter of its fatty acid into the brain crossing the blood-brain barrier with the help of major facilitator superfamily domain-containing 2A receptor [30]. Along with the previous findings, the present study results assume that serum LPA or LPC levels could not be the applicant marker for MDD.

Finally, we measured correlation coefficients between serum LPA or LPC levels with patient characteristics and clinical severity scores of depressions. Here we observed no significant relationships between either serum LPA or LPC levels and patient profile such as age, gender, BMI, education, occupation, economic impression, and smoking history. We did not find any significant positive or negative correlation between serum LPA or LPC levels and the severity of depression. The present study results suggest that serum LPA or LPC levels would not play a significant role as a biomarker for clinical diagnosis of major depression.

This present study has a few limitations. The case-control study and the small sample size might also intervene with the findings. We didn't evaluate the food habit and treatment outcomes on the analyzed parameters. Therefore, we recommend further studies examining the correlation between LPA or LPC signaling systems to discover the role of phospholipids on the pathophysiology of depression.

## Conclusions

The present study concluded that serum LPA and LPC levels would not serve as risk assessment markers for MDD. Serum levels of these lipids did not show any changes in MDD patients compared to HCs and also failed to show any correlation with the severity of depression. As a result, the present study suggests the association between serum LPA or serum LPC where the pathophysiology of depression is not well established. Therefore, this is a preliminary study. We recommend further researches to investigate the exact correlation between targeted glycerophospholipids and depression.
